# Effect of Different Tolerable Levels of Constitutive *mcr-1* Expression on Escherichia coli

**DOI:** 10.1128/spectrum.01748-22

**Published:** 2022-08-18

**Authors:** Han Qiao, Jie Yu, Xiukun Wang, Tongying Nie, Xinxin Hu, Xinyi Yang, Congran Li, Xuefu You

**Affiliations:** a Beijing Key Laboratory of Antimicrobial Agents, Institute of Medicinal Biotechnology, Chinese Academy of Medical Sciences and Peking Union Medical College, Beijing, China; b National Institutes for Food and Drug Control, Beijing, China; University of Pittsburgh

**Keywords:** tolerable *mcr-1* expression, colistin resistance, fitness cost

## Abstract

To study the effect of different tolerable levels of constitutive *mcr-1* expression on Escherichia coli, and to provide direct evidence for moderate resistance mediated by *mcr-1*, construction of E. coli strains carrying *mcr-1* on the chromosome with promoters of different strengths was conducted using λ-red recombination. Our results demonstrated that over-high expression of *mcr-1* cannot be tolerated, and seven constructs with more than 200-fold *mcr-1* transcriptional expression differences were obtained. The colistin MICs of the seven strains increased with the increase of MCR-1 levels, and the highest MIC was 8 μg/mL. Lower expression of *mcr-1* didn’t demonstrate many effects on bacteria, while higher tolerable expression of *mcr-1* tended to show fitness costs in growth rate, competitive ability, and cell structures, but no obvious change of virulence was observed in mice. Bacteria demonstrated colistin MICs of 4–8 μg/mL at *mcr-1* expression levels similar to clinical isolates, which were the *mcr-1* expression levels with relatively lower fitness costs.

**IMPORTANCE** The effects of relatively lower tolerable levels of *mcr-1* were not evaluated thoroughly, and direct evidence for moderate resistance mediated by *mcr-1* was lacking. In the present study, we made constructs carrying *mcr-1* on the E. coli K12 chromosome under the control of serial constitutive promoters of different strengths and studied the effects of different tolerable levels of *mcr-1* expression *in vitro* and *in vivo*. The results demonstrated that generally, except QH0007 (the construct with the highest *mcr-1* expression that showed some extent of cell death), the fitness costs of tolerable *mcr-1* expression on bacteria were not apparent or low. Bacteria demonstrated colistin MICs of 4–8 μg/mL at *mcr-1* expression levels similar to clinical isolates, which corresponded to the lower levels of *mcr-1* expression that can lead to colistin resistance, indicating the cleverness of bacteria to balance the benefit and cost of MCR-1-mediated colistin resistance.

## INTRODUCTION

Polymyxins are a group of nonribosomally synthesized, cationic, cyclic peptide antibiotics discovered in 1947 (1). The mechanism by which polymyxins (polymyxin B and colistin) kill Gram-negative pathogens relies on disruption of membrane permeability through polar and hydrophobic interactions (2). Of these interactions, an electrostatic interaction is present between positively charged residues of polymyxins and the negatively charged lipid A moieties of lipopolysaccharides (LPS) anchored on the outer leaflet of the bacterial membrane. Potential nephrotoxicity and neurotoxicity of polymyxins hamper their wide introduction into regular clinical therapies. However, with the emergence of carbapenem-resistant bacteria (especially in Pseudomonas aeruginosa, Acinetobacter baumannii, Klebsiella pneumoniae and Escherichia coli), polymyxins have become the last line of defense against drug-resistant Gram-negative bacterial infections worldwide.

With increased use in clinics, animal husbandry, and agriculture, the therapeutic effect of colistin on Gram-negative bacteria has been relatively reduced, and colistin resistance emerged. One of the most worrisome is the mobile colistin resistance gene *mcr-1* (usually plasmid borne), first discovered in animal-derived E. coli in 2015 (3). The inner membrane protein MCR-1 can catalyze the transfer of phosphoethanolamine (PEA) to lipid A, resulting in colistin resistance (4). Colistin MICs displayed by *mcr-1*-positive E. coli are moderate (usually 2–8 μg/mL) compared to the level of colistin resistance (usually 8–256 μg/mL) mediated by, for example, increased expression of *pmrA*/*pmrB*, inferring that the expression of *mcr-1* (and hence phosphoethanolamine modification of lipid A) is tightly controlled. This, in part, is supported by the report that *mcr-1* was usually carried by plasmids of a relatively low copy number (5).

Several groups have studied the *in vitro* and *in vivo* fitness costs of *mcr-1*-mediated colistin resistance, but with inconsistent results (6–10). These may at least partly relate to the complexity of the plasmid structures, as the plasmid backbone was reported to have strong effects on fitness burden (11). As certain genes in the *mcr-1-*carrying plasmid may compensate for the fitness cost in colistin-resistant strains, the fitness cost of a plasmid may be different according to plasmid type and bacterial host (10). The fitness of high expression levels of *mcr-1* was studied by induction of an overexpressing plasmid carrying *mcr-1*, and decreased growth rate, cell viability, competitive ability, and significant degradation in cell membrane and cytoplasmic structures were found (12). However, the effects of relatively lower tolerable levels of *mcr-1* were not evaluated thoroughly, and direct evidence for moderate resistance mediated by *mcr-1* was lacking.

Here, we made constructs carrying *mcr-1* on the E. coli K12 chromosome under the control of serial constitutive promoters of different strengths and studied the effects of different tolerable levels of *mcr-1* expression *in vitro* and *in vivo* in aspects of cell growth rate, antibiotic susceptibility, competitive ability, cell membrane and cytoplasmic structures, and virulence. The results will provide more information for understanding how bacteria manage to benefit from *mcr-1* expression with lower costs, and why usually moderate resistance was mediated.

## RESULTS

### Strain construction and *mcr-1* transcriptional expression levels.

Isogenic strains were constructed with *mcr-1* transcriptionally fused to each of the seven constitutive promoters of different strengths on the chromosome. The strains were named QH0001 (J23213), QH0002 (J23112), QH0003 (J23113), QH0004 (J23117), QH0005 (J23115), QH0006 (J23105) and QH0007 (J23110), respectively, with the corresponding promoters listed in the parentheses. The transcriptional expression levels of *mcr-1* varied over a 200-fold range as measured by qPCR, with the strongest promoter expressing *mcr-1* about 186-fold the level of the control genes (Table 1). The three clinical *mcr-1*-carrying isolates, i.e., E. coli 08–85, E. coli 13–66, E. coli 13–43, demonstrated *mcr-1* transcriptional expression of 17- to 34-fold of the control genes, corresponding to levels between QH0004 and QH0005 in the serial constructed strains (Table 1).

**TABLE 1:** Strain characteristics as a function of *mcr-1* transcription level^*a*^

Strains^*b*^	Description	Promoter sequence	mRNA level^*c*^ Mean ± SD	Relative growth rate^*d*^ Mean ± SD	Colistin MICs^*e*^	Protein level^*f*^ Mean ± SD	NPN uptake [%]^*g*^ Mean ± SD
K12 (DA5438)	MG1655			1.00 ± 0.01	0.125	0.04 ± 0.02	0 ± 0.72
QH0001	J23213-*mcr-1*	5′-CTG ATG GCT AGC TCA GTC CTA GGG ATA GTG CTA GC-3′	0.7 ± 0.22	0.98 ± 0.01	0.25	0.34 ± 0.06	−0.35 ± 0.34
QH0002	J23112-*mcr-1*	5′-CTG ATA GCT AGC TCA GTC CTA GGG ATT ATG CTA GC-3′	1.4 ± 0.78	0.98 ± 0.01	0.5	0.48 ± 0.18	0.23 ± 0.41
QH0003	J23113-*mcr-1*	5′-CTG ATG GCT AGC TCA GTC CTA GGG ATT ATG CTA GC-3′	2.5 ± 0.69	0.98 ± 0.02	1	0.51 ± 0.42	0.13 ± 0.54
QH0004	J23117-*mcr-1*	5′-TTG ACA GCT AGC TCA GTC CTA GGG ATT GTG CTA GC-3′	10.0 ± 2.37	0.98 ± 0.01	4	0.83 ± 0.45	0.12 ± 0.63
QH0005	J23115-*mcr-1*	5′-TTT ATA GCT AGC TCA GCC CTT GGT ACA ATG CTA GC-3′	102.6 ± 66.05	0.97 ± 0.01	8	1.29 ± 0.27	0.86 ± 0.35
QH0006	J23105-*mcr-1*	5′-TTT ACG GCT AGC TCA GTC CTA GGT ACT ATG CTA GC-3′	177.8 ± 96.50	0.98 ± 0.01	8	3.14 ± 0.57	0.90 ± 0.14
QH0007	J23110-*mcr-1*	5′-TTT ACG GCT AGC TCA GTC CTA GGT ACA ATG CTA GC-3′	185.7 ± 34.28	0.88 ± 0.02	8	7.96 ± 2.64	16.69 ± 0.36
E. coli 08-85	*mcr-1* carrying	NA	33.96 ± 11.74	ND	4	ND	ND
E. coli 13-66	*mcr-1* carrying	NA	24.76 ± 8.53	ND	8	ND	ND
E. coli 13-43	*mcr-1* carrying	NA	16.95 ± 3.63	ND	8	ND	ND

a NA, not applicable; ND, not determined.

b QH0001 to QH0007 are constructs isogenic to MG1655 with the *mcr-1* gene being placed downstream of J23-series promoters in the *galK* locus. E. coli 08–85, E. coli 13–66, and E. coli 13–43 are clinical *mcr-1*-carrying strains.

c mRNA levels of *mcr-1* relative to control genes *hcaT* and *cysG*, with standard deviations.

d Exponential growth rates relative to E. coli K12 with standard deviations; the experiment was conducted three times on different days, and representative results from one experiment are shown.

e MICs of colistin against E. coli 08–85, E. coli 13–66, and E. coli 13–43 were determined previously (13).

f MCR-1 protein levels quantified by western blot band intensity analysis with a densitometer (*n* = 3).

g NPN uptake rates with 0.1% Triton X-100-treated cells set as the 100% NPN uptake control.

### MCR-1 detection by Western blotting.

**FIG 1:**
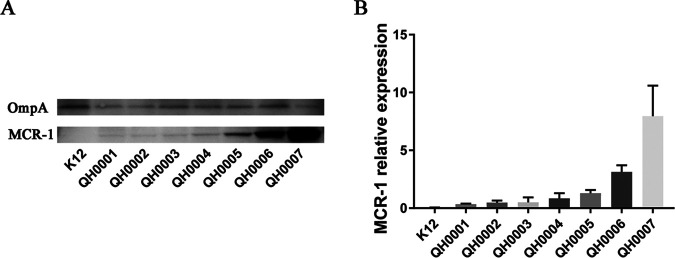
Western blot detection of MCR-1 protein levels in the *mcr-1* constructs. (A) Western blot image for MCR-1 protein detection; (B) MCR-1 band intensity quantification using a densitometer. The experiment was repeated three times; the representative image is shown in panel A, and the MCR-1 quantification results from the three experiments are shown in panel B.

The MCR-1 protein level in each strain was detected by Western blotting with OmpA as the internal control. As shown in Fig. 1, while the control protein OmpA showed similar levels in each strain, the MCR-1 levels differed. The parental strain E. coli K12 (without *mcr-1*) showed no band in MCR-1 detection, and the *mcr-1*-carrying strains (QH0001–QH0007) demonstrated gradually increased MCR-1 protein levels with the increase of promoter strength. The band intensity results analyzed with a densitometer showed that with the increase of the promoter activity, *mcr-1* expression levels increased from 0.34 in QH0001 to 7.96 in QH0007 (Table 1).

### Susceptibility of the strains to different antibiotics.

The broth microdilution method was used to determine the MICs of different antibiotics against the strains (Table 2). The MICs of colistin and polymyxin B increased as the expression of *mcr-1* increased, the highest MICs of the two antibiotics were 8 μg/mL in the serial strains. However, the MICs of aztreonam, ceftazidime, gentamicin, and rifampin showed no apparent change as the expression of MCR-1 increased.

**TABLE 2:** MICs of compounds against strains expressing different levels of *mcr-1*

Compounds	ATCC 25922	MIC (μg/mL) against different E. coli^*a*^							
		K12	QH0001	QH0002	QH0003	QH0004	QH0005	QH0006	QH0007
Colistin	0.25	0.125	0.25	0.5	1	4	8	8	8
Polymyxin B	0.5	0.25	0.25	0.5	1	2	4	8	4
Aztreonam	0.06	0.125	0.125	0.25	0.125	0.125	0.25	0.125	0.125
Ceftazidime	0.25	0.25	0.25	0.5	0.25	0.5	0.5	0.25	0.25
Gentamycin	0.25	0.25	0.25	0.25	0.25	0.5	0.25	0.25	0.5
Rifampin	8	8	16	8	8	8	8	8	8

a MICs were determined in CAMH broth by microdilution methods according to CLSI guidelines.

### Determination of exponential growth rates.

In order to determine whether the expression of *mcr-1* has fitness costs on bacterial growth, Bioscreen was used to determine the growth rates of strains expressing different levels of *mcr-1*. As shown in Table 1, QH0001, QH0002, QH0003, QH0004, QH0005 and QH0006 showed no apparent effects on growth rates in comparison to the parental strain, with relative growth rates being 0.97 to 0.98. QH0007 (the one with the strongest promoter in the current study), however, demonstrated significantly lowered growth rate, with a fitness cost (difference of the relative growth rate of QH0007 in comparison to the parental strain) of about 12%, indicating the adverse effect of over-high expression of *mcr-1* on cell growth.

### *In vitro* competition assay.

**FIG 2:**
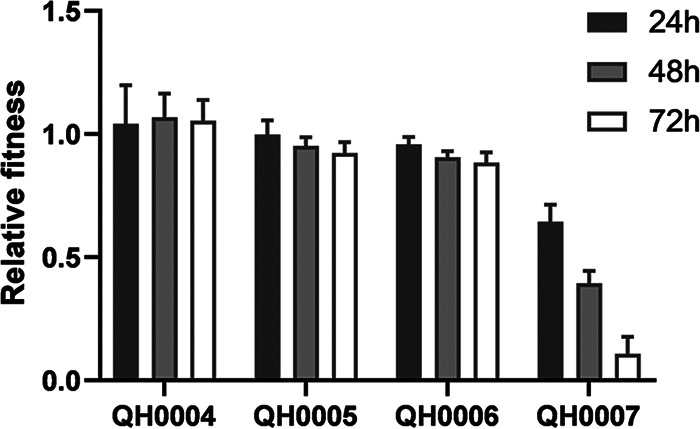
*In vitro* competition assay results of the *mcr-1* constructs versus the parental strain (*n* = 9). The experiments were performed three times on different days with three independent samples each time.

*In vitro* competition assay results (Fig. 2) of the constructs versus parental strain (E. coli K12) demonstrated that QH0004 had a relative fitness of 1.041 to 1.068 at 24 h, 48 h and 72 h, indicating no apparent fitness costs. However, QH0005 (relative fitness of 0.923–0.998), QH0006 (relative fitness of 0.884–0.958), and QH0007 (relative fitness of 0.109–0.645) demonstrated gradually increased fitness costs as the expression level of *mcr-1* increased.

### E. coli morphology observation with SEM and TEM.

**FIG 3:**
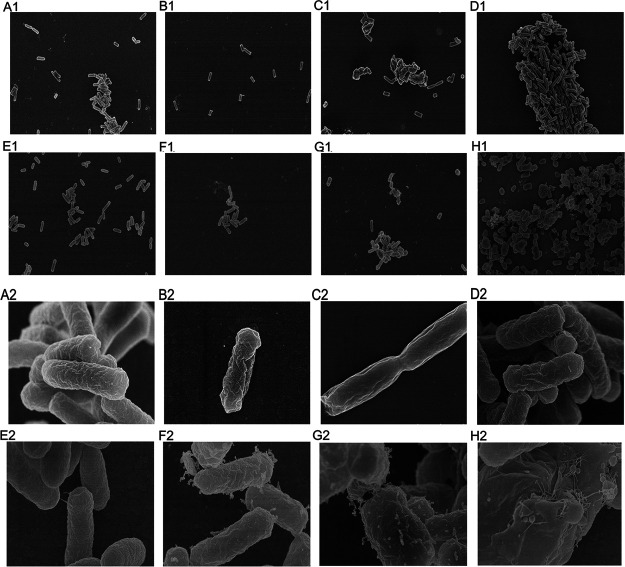
SEM micrographs of E. coli expressing different levels of *mcr-1*. A1–H1: K12, QH0001, QH0002, QH0003, QH0004, QH0005, QH0006, and QH0007 at 4,000-fold enlargement; A2–H2: K12, QH0001, QH0002, QH0003, QH0004, QH0005, QH0006, and QH0007 at 40,000-fold enlargement.

MCR-1 catalyzes the modification of lipid A in the bacterial outer membrane, hence, bacteria expressing MCR-1 may show defects in cellular morphology. We first visualized the morphology of the entire E. coli cells by scanning electron microscopy (SEM). Control cells demonstrated smooth, homogenous cell surfaces and rod-shaped morphology types (Fig. 3A1 and 3A2). QH0001 (Fig. 3B1 and 3B2), QH0002 (Fig. 3C1 and 3C1), QH0003 (Fig. 3D1 and 3D2), and QH0004 (Fig. 3E1 and 3E2) with relatively lower MCR-1 expression showed no significant changes in surface characteristics. QH0005 (Fig. 3F1 and 3F2) had some abnormal secretions on the surface of the cells; QH0006 (Fig. 3G1 and 3G2) demonstrated increased abnormal secretions, a small portion of cell membrane broke, and leakage of intracellular substance was found. The cells of QH0007 (the strain with the highest MCR-1 expression; Fig. 3H1 and 3H2) lost their normal morphology, the abnormal secretions increased, and broken cells were found.

**FIG 4:**
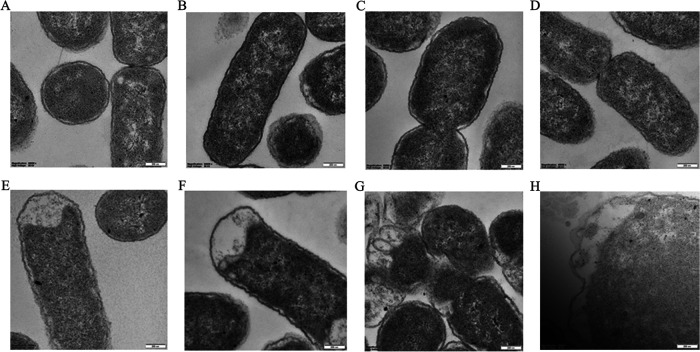
TEM micrographs of E. coli expressing different levels of *mcr-1*. A–H: K12, QH0001, QH0002, QH0003, QH0004, QH0005, QH0006, and QH0007 at 80,000-fold enlargement.

Transmission electron microscopy (TEM) images are shown in Fig. 4. The ultrastructural characteristics of the E. coli K12 cells showed homogeneous cytoplasm and intact cell membrane (Fig. 4A). There were no significant changes in cell membrane and internal structure in QH0001 (Fig. 4B) and QH0002 (Fig. 4C), the strains with relatively weaker promoters. Burr-like folds appeared in the cell membrane structure of QH0003 (Fig. 4D), indicating occurrence of damages. The vacuolar-like structure appeared in the cells of QH0004 (Fig. 4E), and the number of burr-like folds increased. The cell morphology was slightly deformed in QH0005 (Fig. 4F), a vacuolar-like structure appeared, and the cell membrane fold deformation intensified. Cell deformation was more obvious in QH0006 (Fig. 4G), the proportion of cell vacuolar structure increased, and the cell membrane structure became significantly unstable. Cell membrane structures were incomplete in some of the cells of QH0007 (Fig. 4H), and the intracellular structure was blurred.

### Membrane integrity assays.

**FIG 5:**
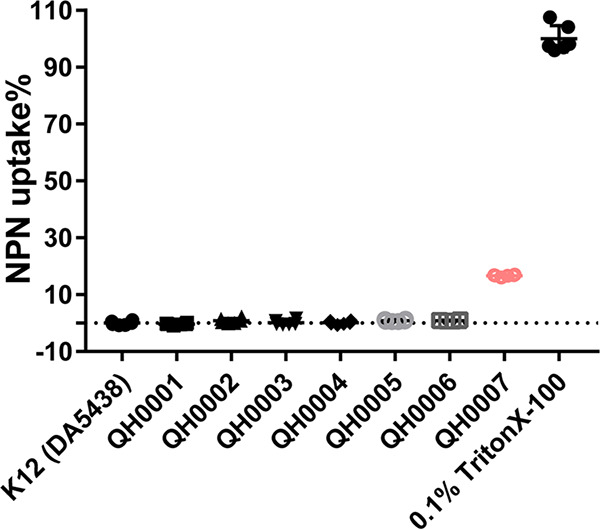
NPN uptake percentages of different strains. Three biological replicates and two technical replicates were used for each strain (six samples for each strain). E. coli K12 (DA5438) treated by 0.1% Triton X-100 was used as the positive control (NPN 100% uptake).

As over-high expression of *mcr-1* posted a significant effect on the cell membrane morphology of E. coli, we further tested the membrane integrity by measuring the 1-N-phenyl-naphthylamine (NPN) uptake rate. As shown in Fig. 5 and Table 1, QH0001, QH0002, QH0003, QH0004, QH0005, and QH0006 demonstrated almost the same levels of NPN uptake as the parental strain; there was no significant difference between these groups and the K12 group (*P* > 0.05 as demonstrated by one-way ANOVA), indicating relatively good integrity of the cell membranes. However, a significant increase in NPN uptake was found in QH0007 (16.69% when 0.1% Triton X-100 treated cells was set as the 100% NPN uptake control; *P* < 0.0001 in comparison to E. coli K12 as demonstrated by one-way ANOVA), suggesting damage on the cell membranes.

### Virulence of different strains to mice.

**FIG 6:**
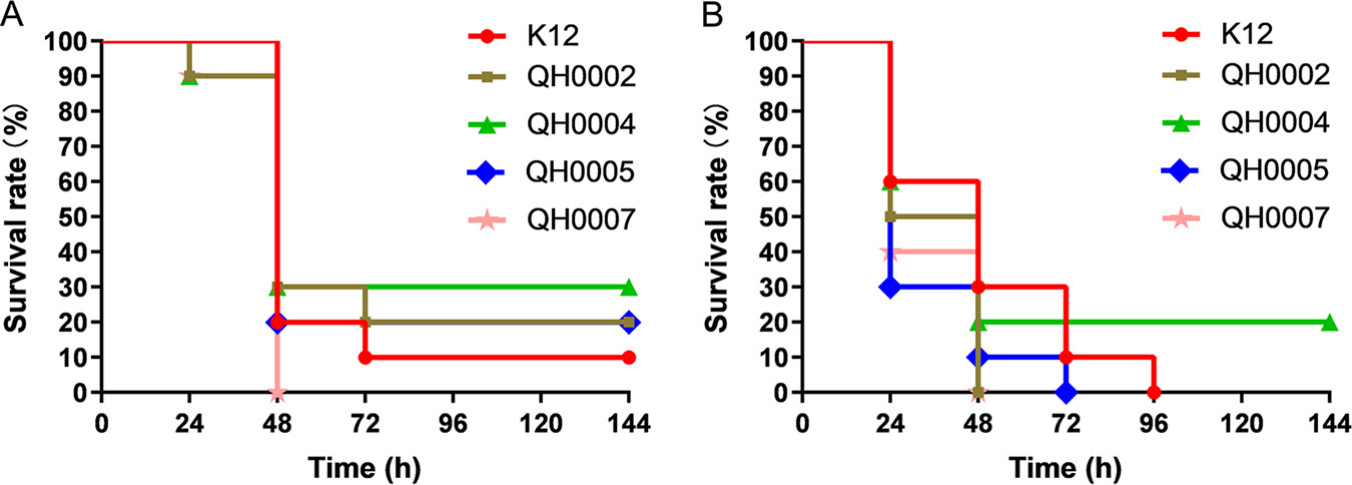
Virulence of strains expressing different levels of *mcr-1*. (A) Mouse systemic infection by intraperitoneal injection in the presence of 5% mucin. Infection doses: E. coli K12, 3.0 × 10^6^ CFU/mouse; QH0002, 3.7 × 10^6^ CFU/mouse; QH0004, 3.3 × 10^6^ CFU/mouse; QH0005, 3.3 × 10^6^ CFU/mouse; and QH0007, 1.9 × 10^6^ CFU/mouse; *n* = 10. (B) Mouse systemic infection by intraperitoneal injection in the absence of 5% mucin. Infection doses: E. coli K12, 1.6 × 10^9^ CFU/mouse; QH0002, 1.8 × 10^9^ CFU/mouse; QH0004, 1.8 × 10^9^ CFU/mouse; QH0005, 1.9 × 10^9^ CFU/mouse; and QH0007, 1.0 × 10^9^ CFU/mouse; *n* = 10.

To assess the effect of different levels of MCR-1 on bacterial virulence, the mouse systemic infection model by intraperitoneal injection was used with strains E. coli K12 (DA5438), QH0002, QH0004, QH0005, and QH0007 as the challenge organisms. In the presence of 5% mucin, the infection doses were 3.0 × 10^6^, 3.7 × 10^6^, 3.3 × 10^6^, 3.3 × 10^6^, and 1.9 × 10^6^ CFU/mouse for E. coli K12 (DA5438), QH0002, QH0004, QH0005 and QH0007, respectively. In the absence of 5% mucin, the corresponding infection doses were 1.6 × 10^9^, 1.8 × 10^9^, 1.8 × 10^9^, 1.9 × 10^9^, and 1.0 × 10^9^ CFU/mouse, respectively. As shown in Fig. 6, no apparent virulence change can be found for the strains with the increase of the *mcr-1* expression in the presence of 5% mucin (Fig. 6A) as well as in the absence of 5% mucin (Fig. 6B).

## DISCUSSION

Decreased growth rate, cell viability, competitive ability, and significant degradation in cell membrane and cytoplasmic structures were found in E. coli by induction of an overexpressing plasmid carrying *mcr-1* (12). However, the effects of lower tolerable levels of *mcr-1*, including *mcr-1* levels found in clinical isolates, were not evaluated thoroughly, and direct evidence for moderate resistance mediated by *mcr-1* was still lacking. In the present study, we conducted construction of isogenic strains with *mcr-1* under the control of constitutive promoters of different strengths at the *galK* locus in the chromosome of E. coli K12. Chromosomal integration avoids complications arising from issues such as plasmid segregation or plasmid maintenance-associated metabolic burden, constant antibiotic selection pressure required for maintenance of plasmids and bacterial artificial chromosomes (BACs) in the cell (14–15).

A total of seven strains were constructed, with *mcr-1* transcriptional expression levels changing over 200 folds as shown by qPCR determination, and the *mcr-1* expression levels were also confirmed by Western blotting (with a range of about 23 folds). The three *mcr-1*-carrying clinical E. coli with colistin MICs of 4 to 8 μg/mL demonstrated *mcr-1* transcriptional expression levels 17- to 34-fold of the control genes by qPCR, corresponding to *mcr-1* levels between those of QH0004 and QH0005 (Table 1). The effects of different tolerable levels of *mcr-1* on bacteria were then studied by growth rate determination, MIC determination, electron microscopy observation, membrane integrity assay, *in vitro* competition assay, and virulence to mice.

MIC results demonstrated that as *mcr-1* expression increased, the MICs of colistin and polymyxin B increased, and the highest MIC that could be obtained was 8 μg/mL, consistent with clinical observations (3). The lower levels of *mcr-1* expression that can confer colistin resistance in the serial strains were those of QH0004 and QH0005 (with colistin MICs of 4–8 μg/mL), which were similar to the *mcr-1* expression levels in the three clinical isolates (Table 1). When a bacterium acquires resistance to an antibiotic, it usually pays a certain cost, i.e., the fitness cost (6, 16). In this study, *mcr-1* expressions in QH0001 to QH0006 had no apparent or lower fitness costs on growth rate, membrane integrity, and susceptibility to other antibiotics. However, in QH0007 with the highest *mcr-1* expression, a fitness cost of 12% was seen in relative growth rate, and dramatic effects on membranes (NPN uptake assay and morphology observation with SEM and TEM) were found. Consistent with the dramatic effects of *mcr-1* expression on cells in QH0007, construction of strains with *mcr-1* expression levels higher than that of QH0007 was not successful in our study (data not shown), indicating that the effects higher than that of QH0007 *mcr-1* expression in cells may be too heavy to be tolerated. *In vitro* competition and electron microscopy results demonstrated the gradual effects of *mcr-1* expression on strains: as the *mcr-1* expression levels increased, the competition strength decreased, and cell morphology and cell structure changed.

It has been reported that colistin resistance may affect bacterial virulence in K. pneumoniae through affecting the amount of polysaccharides (17). Yang et al. (12) also reported highly attenuated virulence of clinical E. coli when transformed with *mcr-1* plasmids in a Galleria mellonella infection model. However, studies from Tietgen et al. (8). stated no substantial effect of *mcr-1* expression on virulence in Galleria mellonella larvae both in E. coli and K. pneumoniae. The inconsistency may lie in the variation of the strains and *mcr-1*-carrying plasmids used (10). To study the effects of pure tolerable *mcr-1* expression on virulence, we used four of the isogenic strains together with E. coli K12 in the mouse systemic infection model by intraperitoneal injection in the presence or absence of 5% mucin. The results demonstrated no apparent virulence changes of the strains, even with the construction of the highest *mcr-1* expression (Fig. 6).

In Gram-negative bacteria, LPS is synthesized in the cytoplasm via the Raetz pathway (18) and introduced into the inner leaflet of the cytoplasmic membrane (CM), which is then flipped to the outer leaflet of the CM by MsbA and transported to the outer membrane (OM) via the LptABCDEFG machinery (19). In the *mcr-1*-carrying isolate (normal *mcr-1* expression level), MCR-1 modifies lipid A with phosphoethanolamine (pEtN) moiety as it passes through the CM on its way to the OM, resulting in most of LPS in the CM modified and colistin resistance. However, a relatively large quantity of unmodified LPS is present in the OM and serves as a target for colistin (20). Under normal expression levels of *mcr-1* (between those of QH0004 and QH0005), there is a sufficient quantity of unmodified LPS in the OM for maintenance of cell integrity, and hence no apparent fitness costs were seen. As the expression level of *mcr-1* increases (from QH0004 to QH0007), the quantity of unmodified LPS in the OM decreases and damage to cell membrane appears (Fig. 3 and 4). When the quantity of unmodified LPS drops to a certain point (lower than that in QH0007), cell membrane damages can be too severe to be tolerated, consistent with our observation that construction of strains with *mcr-1* levels higher than that of QH0007 was not successful. In clinical isolates, bacteria manage to keep the expression of *mcr-1* at the levels (between the levels of QH0004 and QH0005) that can manifest colistin resistance by LPS modification in the CM, while keeping a large quantity of unmodified LPS in the OM for normal function.

In conclusion, our results demonstrated the effects of tolerable pure *mcr-1* expression on bacteria. Generally, except QH0007 (the one with the highest *mcr-1* expression that showed some extent of cell death), the fitness costs of tolerable *mcr-1* expressions on bacteria are not apparent, or low, supporting its wide spread in clinic. Bacteria can demonstrate colistin MICs of 4–8 μg/mL at *mcr-1* expression levels similar to clinical isolates, which correspond to the lower levels of *mcr-1* expression that can lead to colistin resistance, indicating the cleverness of bacteria to balance the benefit and cost of MCR-1-mediated colistin resistance.

## MATERIALS AND METHODS

### Media.

LB broth and LB agar were used as liquid and solid media for bacterial growth. Chloramphenicol was used at 30 μg/mL where indicated, and counter-selection of *sacB* was done on LB agar supplemented with 5% sucrose. BBL TM Mueller-Hinton (MH) II agar (Becton, Dickinson & Company, France) was used for MIC determinations.

### Strains.

E. coli 08–85, E. coli 13–43, and E. coli 13–68 were used as the *mcr-1*-carrying clinical isolates from the Chinese Academy of Medical Sciences Collection Center of Pathogen Microorganisms, CAMS-CCPM-AP. The E. coli K12 (DA5438) strain was obtained from Dan I Andersson of Uppsala University. A set of isogenic strains carrying constitutive promoters of different strengths upstream of a *cat–sacB* cassette at the *galK* locus in E. coli K12 (21) was used for construction of strains with *mcr-1* expressing at different tolerable levels; the promoter sequences were listed in Table 1. The coding sequence for *mcr-1* was amplified from a *mcr-1*-carrying clinical isolate (E. coli 08–85), and transcriptionally fused by recombineering each of the promoters by replacing the *cat–sacB* cassette using methods listed in literature (22). PCR and DNA sequencing (Invitrogen) were used to confirm the genetic constructions. The resulting strains were named QH0001, QH0002, QH0003, QH0004, QH0005, QH0006, and QH0007. The primers used were as follows: *mcr-1* amplification primers, *mcr1*-galK-FP: 5′-AGC AGT ACT GTT ACT AGA GAA AGA GGA GAA ATA CTA GAT GAT GCA GCA TAC TTC TGT GTG-3′, *mcr1*-galK-RP: 5′-AAC GCA AAA AGC CCC GAG CGG TTA AAC TCA GGG CTT TAT TTT TAT CAG CGG ATG AAT GCG GTG CGG TC-3′; Insertion check primers, sYFP-FP: 5′-CAT GGA TCA GCT AAT TTC CG-3′, galk-RP: 5′-TTG TAT TCG CTG CCA ACC AG-3′.

### Laboratory animals.

CD-1 (ICR) mice (18–21 g, half male, half female) were purchased from Vital River Laboratories (Beijing, China). All animals were housed under controlled humidity (30–70%) and temperature (22 ± 3°C), and a 12 h light-dark cycle. Animals had free access to food and water during the study. All the animal studies complied with the animal husbandry guidelines, and all animal experiments were performed according to national standards for laboratory animals in China (GB/T 35892-2018) (23), with approval from the Laboratory Animal Welfare and Ethics Committee in Institute of Medicinal Biotechnology, Peking Union Medical College.

### RNA extraction and qPCR.

RNA extractions were made in three biological replicates. RNA was isolated with cultures of OD_600_ at 0.3–0.4 using an RNAPrep Pure Cell/Bacteria kit (TIANGEN, cat no. DP430) and quantified using a NanoDrop ND-1000 spectrophotometer. FastQuant RT kits (with gDNase) (TIANGEN, cat no. KR106) were used to synthesize cDNA. Quantitation of the mRNA levels for the *mcr-1* was performed on the 7500 Fast real-time PCR system (Applied Biosystems) with the PowerUp SYBR green master mix (Applied Biosystems) according to the manufacturer’s instructions. Primers for RT-PCR were synthesized by Sangon Biotech (Shanghai). PCR amplification was carried out in a total volume of 20 μL, containing 8 μL 1:10, 1:100, 1:1000, and 1:10000 diluted cDNA solution, 10 μL of 2 × PowerUp SYBR green master mix, and 1 μL of each primer. Control housekeeping genes were *cysG* and *hcaT*. Primers used were as follows: qPCR-*mcr1*-FP: 5′-GCT CCA AAA TGC CCT ACA GA-3′; qPCR-*mcr1*-RP: 5′-CTT GGT AGC ACA CCC AAA CC-3′; qPCR-*cysG-F*P: 5′-TTG TCG GCG GTG GTG ATG TC-3′; qPCR-*cysG* –RP: 5′-ATG CGG TGA ACT GTG GAA TAA ACG-3′; qPCR-*hcaT*-FP: 5′-GCT GCT CGG CTT TCT CAT CC-3′; qPCR-*hcaT*-RP: 5′-CCA ACC ACG CTG ACC AAC C-3′.

### Western blot analysis.

A total of 4–5 single colonies were inoculated in 3 mL LB medium and incubated at 37°C overnight. Cell pellets from 2 mL of overnight cultures were collected by centrifugation at 8,000 rpm for 10 min, washed three times with physiological saline, and resuspended in 200 μL of radioimmunoprecipitation (RIPA) lysate (Beyotime, cat no. P0013C). The cell suspensions were sonicated for 1.5 min (work 3 s, stop 6 s, with frequency of 28 KHz), centrifuged at 14,000 rpm, 4°C for 10 min, and the supernatants were collected. Equal amounts of proteins (100 μg/lane) from each bacterial lysate were separated by SDS-PAGE and transferred to polyvinylidene difluoride membranes (Hybond-P; GE Healthcare). Blots were probed with the Polymyxin resistance protein MCR-1 primary antibody (polyclonal mouse anti-E. coli polymyxin resistance protein MCR-1 antibody; LS Bio), followed by horseradish enzyme labeled goat antimouse IgG (ZB-2305; ZSGB-Bio). Protein bands were visualized using an enhanced chemiluminescence (ECL) detection method (Bio-Rad), and band intensities were analyzed with a densitometer (LAS-4000; GE Healthcare). The experiment was repeated 3 times. OmpA, measured quantitatively using an OmpA antibody (polyclonal rabbit anti-Salmonella typhi OmpA antibody; LS Bio), followed by horseradish enzyme labeled goat anti-rabbit IgG (ZB-2301; ZSGB-Bio), was used as the control.

### MIC determination.

The MICs of the antibacterial agents for all constructed E. coli isolates were determined by the broth microdilution method according to CLSI guidelines (24). The final inoculum in each well was about 5 × 10^5^ CFU/mL. The microtiter plates were incubated at 37°C for about 18 h, and the results were recorded by naked eyes. MICs were defined as the lowest concentrations of antibiotics that can inhibit the visible growth of the tested organisms.

### Measurement of growth rates.

The exponential growth rates of E. coli K12 and the *mcr-1*-carrying constructs were measured in MH broth at 37°C by taking optical density at 600 nm (OD_600_) every 4 min in a Bioscreen C reader (Oy Growth Curves Ab Ltd.) (25). Four independent cultures per strain were grown overnight until saturation. The cultures were diluted 1,000-fold and aliquoted into a Bioscreen C plate in duplicate (0.3 mL/well). The growth rates were estimated from the OD_600_ interval between 0.01 and 0.1, where the growth was observed to be exponential. Relative growth rates of the strains were calculated by comparing the growth rates with that of E. coli K12.

### Competition experiments *in vitro*.

To assess the fitness cost of different *mcr-1* expression levels, the four *mcr-1*-carrying strains with relatively strong promoters (QH0004, QH0005, QH0006, and QH0007, which showed high colistin MICs that can be selected out on plates containing 2 μg/mL colistin) were subjected to *in vitro* competition assay against E. coli K12. Strains were cultured in LB broth overnight, after that, strain cultures were adjusted to the 0.5 McFarland standard and 10-μL aliquots of each competitor was mixed with E. coli K12 at a 1:1 ratio. The mixtures were then inoculated in 10 mL LB broth, and incubated at 37°C with shaking, the mixed population was diluted 1,000-fold into fresh LB broth every 24 h until the competition experiment had lasted for 72 h. The total number of bacteria- and colistin-resistant cells were determined by spreading properly diluted samples of each competition mixture on nonselective (without colistin) and selective (with 2 μg/mL colistin) LB agar plates at 0, 24, 48, and 72 h. The formula RF = (log_10_ S1_t_− log_10_ S1_0_)/(log_10_ S2_t_− log_10_ S2_0_) (26) was used to calculate the relative fitness (RF), where S1 and S2 represent CFU densities of the constructed isolates and E. coli K12, respectively, and t is the measure time in hours. If there exists a fitness cost between the competitors, then RF<1; if not, RF>1.

### Morphology observation with scanning electron microscopy (SEM) and transmission electron microscopy (TEM).

The morphology of E. coli strains with different levels of *mcr-1* expression was observed by TEM and SEM. E. coli strains were grown in cation-adjusted Mueller-Hinton (CAMH) broth overnight at 37°C; 2 mL of the stationary-phase cell cultures were collected and fixed with 2.5% glutaraldehyde for at least 24 h at 4°C. For SEM (27), the samples were then centrifuged to remove glutaraldehyde and resuspended in phosphate buffer. The bacterial suspensions were spotted on a polished silicon wafer and dried overnight in a biosafety cabinet. After drying, the samples were coated with chromium and subjected to SEM imaging using Hitachi-SEM-SU8010. For TEM (12), the fixed organisms were washed and further fixed with 1% osmium tetroxide. The samples were washed, dehydrated in a graded series of ethanol, and embedded in Epon Araldite. Ultrathin sections containing the cells were then placed on copper grids, stained with uranyl acetate and lead citrate, observed, and photographed with a JEM-1400 plus. 

### Outer membrane integrity assays.

Overnight cell cultures were diluted 50-fold in fresh LB medium and incubated until midlog phase (OD_600_ of about 0.5). The cell cultures were then centrifuged, the pellets were washed 3 times with 5 mM HEPES buffer containing 20 mM glucose (pH 7.2) and resuspended in the same buffer. Then, 100-μL cell suspensions were then mixed with 100 μL of the 20 μM 1-N-phenyl-naphthylamine (NPN) solution (prepared in the same buffer as the cell suspensions) in each well of the 96-well black clear-bottom plates. Fluorescence was read immediately (within 3 min) in an EnSpire 2300 (excitation wavelength 355 nm, emission wavelength 420 nm). Three biological replicates and two technical replicates were used for each strain. E. coli K12 (DA5438) treated by 0.1% Triton X-100 was used as the positive control. Percent NPN uptake was calculated for each strain as previously described (3, 28):

$$\text{NPN uptake }(\%)=\frac{\text{Fobs}-\text{F}0}{\left(\text{F}100-\text{F}0\right)}*\text{1}00\%$$

where Fobs is the observed fluorescence of a given strain, F0 is the initial fluorescence of NPN with E. coli K12 (DA5438) without treatment, and F100 is the fluorescence of NPN with E. coli K12 (DA5438) upon addition of 0.1% Triton X-100.

### Murine systemic infection model.

E. coli strains K12, QH0002, QH0004, QH0005, and QH0007 were subjected to *in vivo* virulence comparison using the murine systemic infection model by intraperitoneal injection in the presence or absence of 5% mucin. For infections in the presence of 5% mucin, cell pellets from fresh overnight cell cultures were first washed and resuspended in saline (adjusted to 0.5 McFarland), then further diluted 10-fold in 5% mucin. Then, 0.5 mL of the bacterial suspensions in 5% mucin were injected intraperitoneally to each mouse randomly allocated to different groups with a final infection dose of 2–4 × 10^6^ CFU/mouse. Animal deaths were recorded at 24, 48, 72, 96, 120, and 144 h postinfection, and the cumulative survival rates for each time point were calculated. For infections in the absence of 5% mucin, cell pellets from fresh overnight cell cultures were washed in saline, and then resuspended in saline and adjusted to 50 McFarland; 0.2 mL of the bacterial suspensions were then injected intraperitoneally to each mouse randomly allocated to different groups with a final infection dose of 1–2 × 10^9^ CFU/mouse. Animal deaths were recorded at 24, 48, 72, 96, 120, and 144 h postinfection, and the cumulative survival rates for each time point were calculated.
